# Material-Induced Platelet Adhesion/Activation and Hemolysis of Membrane Lung Components from Extracorporeal Membrane Oxygenation

**DOI:** 10.3390/biomedicines13102323

**Published:** 2025-09-23

**Authors:** Christopher Thaus, Matthias Lubnow, Lars Krenkel, Karla Lehle

**Affiliations:** 1 Department of Cardiothoracic Surgery, University Hospital Regensburg, 93053 Regensburg, Germany; christopher.thaus@stud.uni-regensburg.de; 2 Department of Internal Medicine II, University Hospital Regensburg, 93053 Regensburg, Germany; matthias.lubnow@ukr.de; 3 Regensburg Center of Biomedical Engineering, Ostbayerische Technische Hochschule, 93053 Regensburg, Germany; lars.krenkel@oth-regensburg.de

**Keywords:** ECMO, platelet activation, platelet adhesion, hemolysis, thrombosis

## Abstract

**Background:** Contact between blood and the large artificial surfaces within membrane lungs (MLs) is one reason for device-induced thrombus formation during extracorporeal membrane oxygenation (ECMO). **Methods:** Hemocompatibility testing of gas-exchange fibers (GFs) and heat-exchange fibers (HEs) from commercially available/non-used MLs (ML-type, coating: PLS, Bioline^®^; Hilite7000LT, X.ELLENCE^®^; Nautilus, Balance^®^; EOS, PH.I.S.I.O^®^) included static hemolysis and platelet adhesion/activation assays. Platelet activation of non-adherent platelets was identified after antibody (CD62P, PAC-1, CD61) and fibrinogen staining (flow cytometry). The surface coverage (%) of adherent platelets was quantified after F-actin filament-staining. **Results:** All materials were non-hemolytic and did not induce platelet activation. However, platelet adhesion (median (IQR)) depended on the type of surface coating of GFs made entirely of polymethylpentene. Both uncoated GFs (12 (7–19)%) and X.ELLENCE-coated GFs (Hilite-ML, 13 (8–19)%) showed a significantly higher surface coverage compared to Balance-coated GFs (Nautilus-ML, 3 (1–6)%), PH.I.S.I.O-coated GFs (EOS-ML, 2 (2–5)%) and Bioline-coated GFs (PLS-ML, 4 (1–8)%) (*p* < 0.001). HEs made of polyethyleneterephthalate (Hilite-ML, Nautilus-ML) that were coated with X.ELLENCE were covered with more platelets (5 (3–7)%) compared to Balance-coated HEs (3 (1–6)%), respectively (*p* = 0.029). **Conclusions:** In vitro testing disclosed fourfold higher platelet adhesion on X.ELLENCE-coated GFs (and HEs) from the Hilite-ML compared to other ECMO-materials. Additional hemocompatibility tests are necessary to assess the increased platelet adhesion on the materials from the Hilite-ML.

## 1. Introduction

Extracorporeal membrane oxygenation (ECMO) is used to manage critically ill patients with severe respiratory and cardiac failure [1]. Despite improvements in technology and the biocompatibility of the device, patients on ECMO remain at high risk of hematologic complications [2,3]. The main reason for clot formation is the blood exposure to the different ECMO circuit’s foreign biomaterials for a long time. The mechanism is mediated by the adhesion of plasma proteins to the membrane surface and induction of platelet adhesion/activation (mediated via CD62P, glycoprotein IIb/IIIa, fibrinogen binding) leading to the activation of the patient’s inflammatory response and coagulation [4,5,6,7]. As a consequence, the risk of device-induced clot formation, particularly within the membrane lung (ML) increased and required life-threatening device replacements, with a frequency of 3 to 26% [8,9]. This is despite systemic anticoagulation and antithrombotic surface coatings [10]. The latter are offered in bioactive and biopassive configurations [11]. Biopassive coatings aim to reduce interactions between blood and foreign surfaces by inhibiting protein adsorption, while bioactive coatings act specifically by inhibiting coagulation factors, platelets or other components involved in coagulation or hemostasis [10]. Commercially available coatings such as Bioline^®^ (Getinge) or X.ELLENCE^®^ (Fresenius Medical Care) use albumin as part of a multilayer, bioactive coating in alternating layers with heparin to combine the passivation effect of albumin and the antithrombin-III (ATIII)-mediated inactivation of Factors Xa and IIa of heparin [10,11]. In contrast, biopassive coatings with phosphorylcholins (PHISIO^®^, LivaNova) or Balance^®^ Biosurface (Medtronic) provide an antiadhesive surface [10]. PHISIO-coating mimics the nonthrombogenic outer lipid component of natural cell membranes of erythrocytes and endothelial cells and exerts antithrombogenic activity via reducing platelet activation and protein adsorption. Balance Biosurface is claimed to mimic the vascular endothelium with polyethyleneoxide (PEO) and sulfate/sulfonate groups. PEO is a hydrophilic polymer creating an insulating water layer structure between the blood and artificial surface to resist cell adhesion and protein deposition. Sulfate/sulfonate groups not only provide negative charges to repel platelets but also inhibit thrombin by binding to ATIII in a heparin-like manner and may impair additional processes required for thrombus formation (details see [10]).

Regarding the antithrombogenic efficacy of commercially available coatings, so far, there has been no robust evidence to demonstrate that any non-heparin coating is superior to the surface-bound heparin coatings [10]. In this context, the blood compatibility of surface coatings is essential and based primarily on an increased resistance to plasma protein adsorption and platelet adhesion predominantly by a steric repulsion mechanism [6,7,12]. These studies mainly used dynamic hemocompatibility tests (e.g., the Chandler loop and the ECMO thrombosis-on-a-chip models) [10,13,14,15,16] or settings of cardiopulmonary bypass rather than ECMO [10] to demonstrate the response of whole blood on the biomaterial/coating and the effect of shear forces. None of the studies allowed a comparison of all ECMO coatings in one experimental approach [10]. In contrast, static culture models were used to demonstrate the isolated material-induced effects of ECMO material coatings on single blood cells [6,7]. Such tests were often part of biocompatibility studies to exclude surfaces with undesirable reactions [17]. A recent study used a static culture model and tested the effects of isolated granulocytes on the different ECMO coatings [18]. Isolated granulocytes adhered regardless of the coating but with a significantly higher formation of neutrophil extracellular traps on uncoated compared to coated materials [18].

Based on the results from Foltan et al. [18], the aim of the present study was to introduce a simple and cost-effective but also meaningful static culture model to compare the coating performance of a multitude of ECMO materials from different commercially available MLs with different bioactive (Bioline, X.ellence) and biopassive (Physio, Balance Biosurface) coatings. The extent of red blood cell (RBC) lysis (hemolysis), platelet adhesion (cytoskeletal staining with rhodamine-phalloidin) and platelet activation (CD62P, glycoprotein IIb/IIIa and fibrinogen binding activity) were analyzed relative to the uncoated base material.

## 2. Materials and Methods

### 2.1. Preparation of Test Materials

Test material (GF, gas-exchange fiber; HE, heat-exchange fiber) was prepared from non-used naïve MLs from different producers (PLS, Getinge, Rastatt, Germany; Hilite 7000LT, Fresenius Medical Care, Bad Homburg, Germany; Nautilus, Medtronic, Meerbusch, Germany; EOS, LivaNova, Munich, Germany). All GFs consisted of polymethylpentene (PMP), while the HEs were made of different materials (Table S1). Each ML from different manufacturers was coated with individual antithrombotic coatings (Table S1). Uncoated GFs (GF-PMP, reference material) were kindly provided by Getinge. Figure S1 illustrates the preparation path, from the whole ML (here an EOS-ML) to the test material. A band saw was used to remove the housing of the MLs. Sawing particles were withdrawn by suction. The internal block (stacked or wrapped mats of GF and HE membranes) was handled under sterile conditions. Test materials were trimmed to a size of 1 cm × 2 cm (hemolysis) or 2 cm × 2 cm (platelet adhesion). These samples were labeled as GF or HE, along with their respective ML (Bioline^®^ coating, GF-PLS, HE-PLS; X.ELLENCE^®^ coating, GF-Hilite, HE-Hilite; PH.I.S.I.O^®^ coating, GF-EOS; Balance^®^ coating, GF-Nautilus, HE-Nautilus). Due to the rigidity of the stainless steel heat exchanger, a flat arrangement of these samples was not possible, so HE-EOS could not be investigated.

Before analysis, the test materials were soaked in 70% ethanol (30 min) and immersed in sterile physiologic saline (0.9% *w*/*v*; NaCl) (3×, 37 °C, 30 min).

### 2.2. Ethics Statement

All participants enrolled in this research gave informed consent. This study was approved by the Ethics Committee from the University of Regensburg (vote No. 16-101-0322), and the protocol was deemed acceptable by committee.

### 2.3. Blood Collection and Preparation of Platelet-Rich Plasma

Whole blood was extracted via venipuncture from aspirin-free healthy adult human donors (n = 7) and was prevented from coagulation with ethylenediamine tetraacetic acid (EDTA, 1.2 mg/mL; 6 mL) or trisodium citrate at a volumetric ratio of 9:1 (30 mL).

For the hemolysis assay, EDTA-anticoagulated blood was diluted with physiologic saline (0.9% *w*/*v*; NaCl) at a ratio of 1:1.25.

For the platelet adhesion/activation assays, citrated blood was stabilized for 30 min at room temperature (RT) with a slight rolling movement. Afterward, blood samples were centrifuged (300× *g*, 10 min, RT). A volume of 1.1 mL of the supernatant (platelet-rich plasma, PRP) was transferred into another sterile tube. The remaining blood sample was again centrifuged (3× 3320× *g*, 10 min, RT). The supernatant was platelet-poor plasma (PPP) and used to dilute the PRP-samples (10%-PRP).

Each test material was incubated in parallel with blood (hemolysis) or 10%-PRP (platelet adhesion/activation) from all donors.

### 2.4. Hemolysis Assay

Non-lysed (NL) and completely lysed (CL) cells were prepared by mixing blood with NaCl or distilled water (4:5), respectively. Each test material was incubated with 800 µL aliquots of diluted EDTA-blood (37 °C, 1 h). The solution of each well was collected and centrifuged (1200× *g*, 10 min). The optical density (OD, 540 nm) of the supernatant represents the relative concentration of free hemoglobin released from RBCs (hemolysis).

The hemolysis rate (%) was calculated as follows:

hemolysis rate (%) = [(OD_sample_ − OD_NL_)/(OD_CL_ − OD_NL_)] × 100

where OD_sample_, OD_NL_ and OD_CL_ represent the OD of the test material samples, of non-lysed cell and of completely lysed cells [19]. According to ISO 10993-4:2017 and ASTM F756-00(2000), a material is considered non-hemolytic if its mean hemolysis rate is <5% [20,21].

### 2.5. Platelet Adhesion Assay

Each test material sample was placed in a 6-well plate, held down with a sterile stainless-steel frame, and incubated with 1.5 mL 10%-PRP (60 min, 37 °C). Each material was incubated with platelets from 7 different blood donors. The supernatant was carefully removed for subsequent flow cytometric analysis (FACS) to calculate material-induced platelet activation. The test material was gently rinsed in phosphate-buffered saline (PBS, pH 7.4) supplemented with bovine serum albumin (BSA, 0.5%) and fixed in paraformaldehyde (final, 1% in PBS) (30 min, RT). Adherent platelets were stained with rhodamine-labeled phalloidin (Invitrogen, Carlsbad, CA, US) (1:200 diluted in PBS) for the F-actin cytoskeleton staining (60 min, RT, protected from light). Samples were mounted between two coverslips (24 mm × 60 mm) in Fluoromount-G (Southern Biotech, Birmingham, AL, USA) (overnight, darkness).

For quantification of adherent platelets (Figure S2), images were acquired with a 40× objective using a fluorescence microscope (BZ-8100E Keyence, Neu-Isenburg, Germany) with an integrated camera system. To estimate the extent of cellular deposits, two consecutive capillaries of the stained sample were selected and digitalized. Images from 5 different fields were imported into the ImageJ Version 1.54p (https://imagej.net/ij/download.html, accessed on 17 September 2025) program (National Institute of Health, Bethesda, MD, USA). Conversion to grayscale was performed to distinguish between areas of single cells or aggregates and background. Individual region of interests (ROI) were selected along the gas fibers. The area of detected particles (platelets) in µm^2^ was calculated using the scale bar on the images. Total area of ROI was 0.77 mm^2^. Preliminary experiments showed a good correlation between automated and manually determined particle counts. Platelet coverage was subdivided into particles < 10 µm^2^, 10–100 µm^2^, and >100 µm^2^. The cutoffs came from the cross-sectional area of single platelets (2–8 µm^2^), small aggregates (10–100 µm^2^) and macro-aggregates (>100 µm^2^). The amount of delimitable particles, the individual particle areas and the sum of the area of all particles within the individual ROIs were determined. Surface coverage (%) was calculated as the proportion of area of all particles relative to total area of ROI. The proportion of different particle sizes relative to the total particle count was calculated.

Statistical comparisons were only meaningful for (1) identical base material (GFs made of PMP; HEs made of polyethyleneterephthalate) to demonstrate the coating effect or (2) identical coatings (GFs and HEs from the same ML) to demonstrate the material effect.

### 2.6. Material-Induced Platelet Activation

Since the present study focused on platelet adhesion on different ECMO materials/coatings, additional expression of activation markers [6] on the surface of adherent platelets was not possible. However, non-adherent cells in the supernatant of the adhesion experiments were available to verify a possible activation of circulating platelets after material contact. In the clinical context, this could be an indicator of systemic inflammation and coagulation activation. PRPs without material contact were stimulated (ADP, 50 µM) and non-stimulated (PBS) for 4 min at RT and used as controls. The cells were incubated with specific antibodies (protected from light): FITC-conjugated mouse anti-human PAC-1 (5 µL; BD Biosciences, San Jose, CA, USA) recognizes an epitope on the glycoprotein (GP) IIb/IIIa complex of activated platelets at or near the platelet fibrinogen receptor (manufacturer’s datasheet). PE-conjugated mouse anti-human CD62P (5 µL; BD Biosciences) recognizes an epitope on the adhesion molecule CD62P, which mediates the adhesion of activated platelets to neutrophils and monocytes in hemostasis (manufacturer’s datasheet). PerCP-Cy5.5-conjugated mouse anti-human CD61 (5 µL; BD Biosciences) recognizes GPIIIa that forms complexes with other plasma protein receptors (CD41, CD51) and appears to bind to plasma proteins to mediate cell adhesion. Furthermore, AlexaFluor488-labeled fibrinogen (AF488, Thermo Fisher, Waltham, MA, USA) (stock solution, 1.5 mg/L, 1.5 µL) identified the fibrinogen-binding activity of platelets [22].

One aliquot of the supernatants, as well as of the controls (100 µL), were incubated with antibodies (PAC-1-FITC, CD62P-PE) (45 min, RT), fixed with equal volumes of paraformaldehyde (2%, 30 min, RT), washed with PBS/0.5% BSA (2 mL), and centrifuged (520× *g*, 10 min, RT). Another aliquot (100 µL) was incubated with fibrinogen-AF488 (4 min, RT), fixed with paraformaldehyde, washed and centrifuged. The cell pellet was resuspended in 100 µL PBS/0.5%BS and stained with the anti-human CD61 antibody (45 min, RT). Subsequently, cells were washed and centrifuged. Each pellet was resuspended in 500 µL PBS/0.5% BSA, measured by a flow cytometer (FACSCalibur, BD Bioscience) and analyzed with the FlowJo data analysis package (Version 7.25, University of Washington, Washington, DC, USA). Platelets stained with PAC-1 and CD62P were identified according to their forward (FSC) and low SSC signal. These cells were divided into PAC1-FITC-positive and/or CD62P-PE-positive (or both) platelet populations. The proportion of positive cells, as well as its median fluorescence intensity (median FI), was analyzed. Fibrinogen-stained platelets were identified by CD61-positive fluorescence and 90 degrees light scatter (SSC). This population was subdivided into non-stimulated (fibrinogen-negative) or stimulated platelets (fibrinogen-positive).

### 2.7. Statistics

Statistical analysis was performed using SigmaStat 3.5 (SYSTAT Software, San Jose, CA, USA). Continuous variables were shown as median (interquartile range, IQR), and categorical variables were expressed as frequencies (percentage). Since the cells from individual blood donors showed a high variability of the absolute values analyzed in this study, we considered the volunteers as a second factor in the statistical analysis. Therefore, a two-way analysis of variance (ANOVA) was used to compare material-induced responses. If significant differences of the material response were detected, post-hoc analyses (Bonferroni) were applied to identify pairwise differences. A *p*-value < 0.05 was considered significant.

## 3. Results

### 3.1. Hemolytic Effect of ECMO Material from Different MLs

Water induced complete RBC lysis (CL). Detection of OD_CL_ was only possible after dilution (1:10) (mean ± standard deviation, 2.4 ± 0.4). Non-lysed RBCs (NL) resulted in a mean OD_NL_ of 0.20 ± 0.09. The contact of blood with the test material samples resulted in a mean OD_sample_ of 0.19 ± 0.09. The calculated mean hemolysis rate of each ECMO material was below 5% (GF-PMP, 0.05 ± 0.06%; GF-PLS, 0.06 ± 0.09%; GF-Hilite, 0.05 ± 0.05%; GF-EOS, 0.06 ± 0.06%; GF-Nautilus, 0.07 ± 0.08%; HE-PLS, 0.07 ± 0.06%; HE-Hilite, 0.06 ± 0.08%; HE-Nautilus, 0.06 ± 0.11%; not significant) and, therefore, all test materials were classified as non-hemolytic [20,21].

### 3.2. Incidence of Platelet Activation of ECMO Material from Different MLs

**Figure 1:**
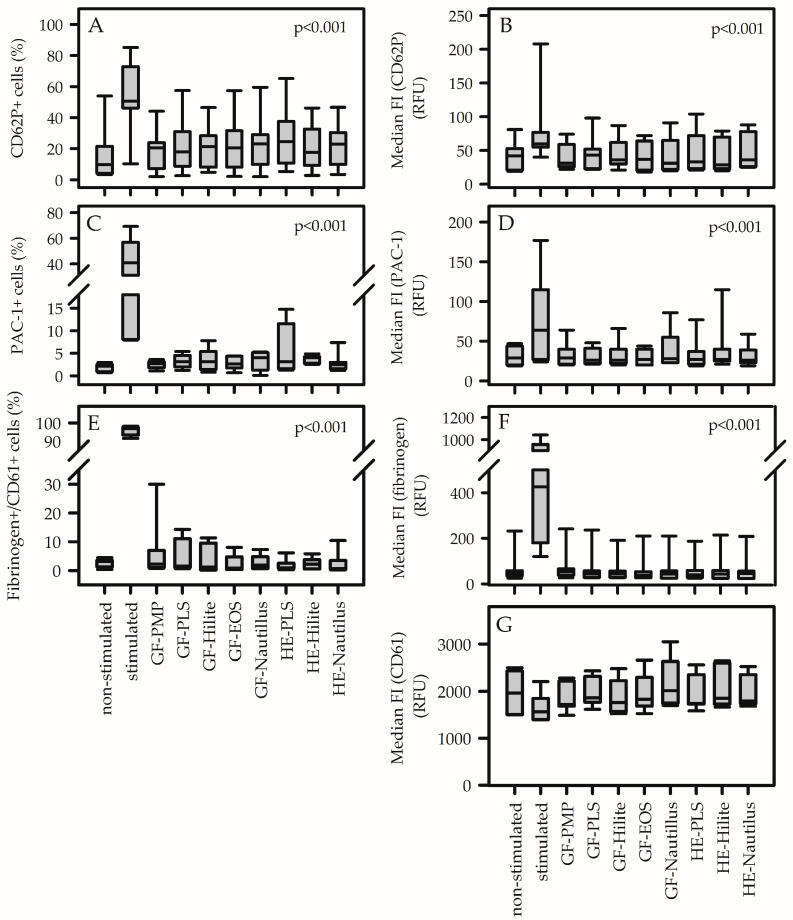
Contact between platelets and ECMO materials did not activate non-adherent platelets with respect to CD62P expression (**A**,**B**), PAC-1 binding (**C**,**D**) and fibrinogen binding to CD61-positive platelets (**E**–**G**). The proportion of CD62P-positive (**A**) and PAC-1-positive (**C**) cells, the median fluorescence intensity (median FI) of CD62P (**B**) and PAC-1 (**D**), the proportion of double-stained platelets (fibrinogen+/CD61+) (**E**) and the median FI of fibrinogen (**F**) were significantly elevated for stimulated cells (without material contact) compared to non-stimulated cells (without material contact) and cells after contact with membrane lung materials (GF, gas-exchange fiber; HE, heat-exchange fiber) from different membrane lungs (PLS, Hilite, EOS, Nautilus). The median FI of CD61 (**G**) was independent of material contact. The manufacturer of the membrane lungs determines the type of antithrombogenic surface coating used (Table S1). RFU represents relative fluorescence intensity. Data are presented as median (IQR). Statistics: two-way ANOVA, including pairwise comparison of each material within each volunteer. *p*-values indicate the difference of each material relative to the stimulated control (relative to the value of the individual volunteer).

The supernatant of platelet adhesion experiments contained non-adherent platelets after material contact. Activation markers on the surface of these cells were identified and quantified as the proportion of positive cells as well as the extent of the respective median fluorescence intensity of bounded antibodies. Figures S3 and S4 show representative FACS analysis of material-induced platelet activation. None of the analyzed ECMO material surfaces induced platelet activation. There was no increase in the proportion of CD62P-positive cells and PAC-1-positive cells (Figure 1A,C). The median fluorescence intensity of respective cell populations remained unchanged (Figure 1B,D). Furthermore, contact between platelets and different ECMO materials did not increase fibrinogen-binding and CD61 expression (Figure 1E–G). Only treatment of platelets with ADP significantly increased platelet activation parameters (except median fluorescence intensity of CD61+ cells) compared to non-stimulated cells and to cells after contact with all ECMO surfaces. *p*-values in Figure 1 demonstrate statistical differences relative to ADP stimulation.

### 3.3. Incidence of Platelet Adhesion of ECMO Material from Different MLs

**Figure 2:**
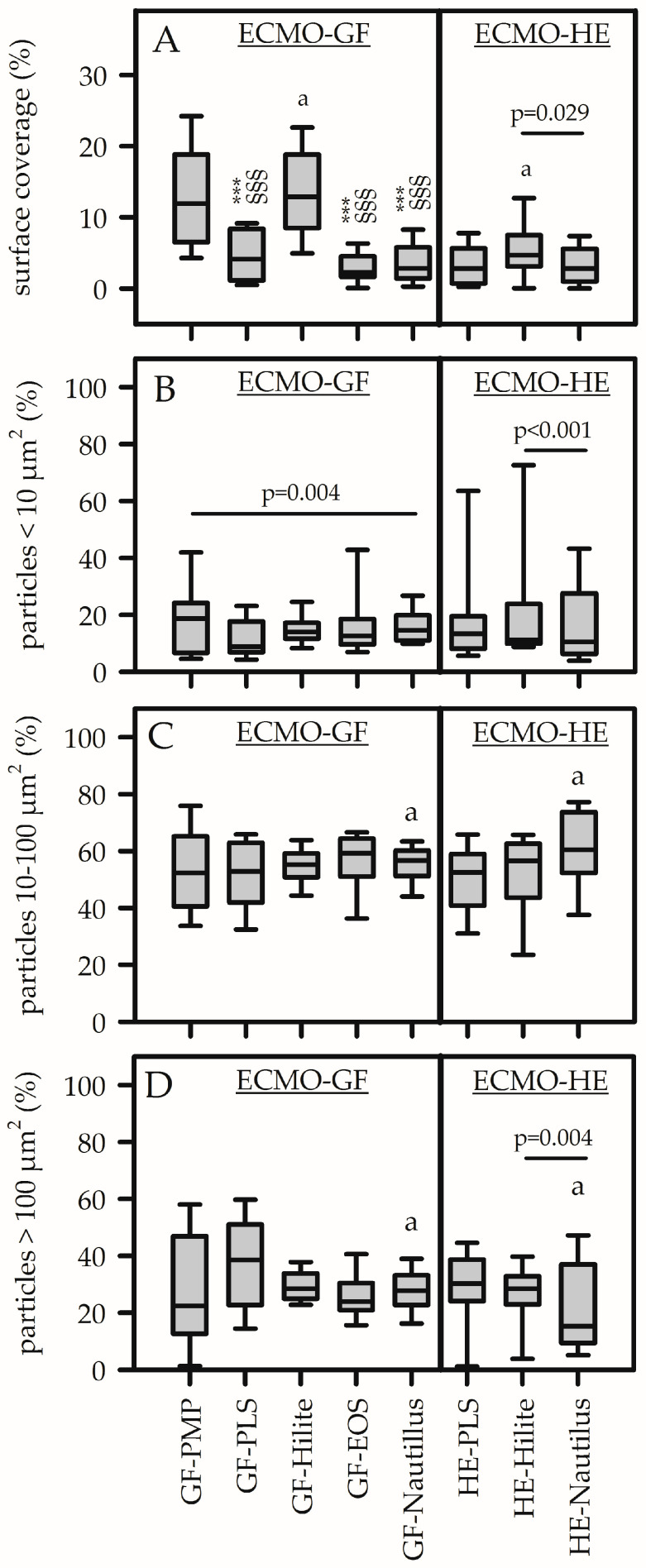
Surface coverage (**A**) and particle size distribution (**B**–**D**) of adherent platelets on different ECMO materials (GF, gas-exchange fiber; HE, heat-exchange fiber) from different membrane lungs (PLS, Hilite, EOS, Nautilus). GF-PMP (PMP, polymethylpentene) was uncoated. Composition of coated GFs and HEs (see Table S1). (**A**) Surface coverage of adherent platelets was significantly higher on GF-PMP and GF-Hilite compared to the GF-PLS, GF-Nautilus and GF-EOS. The distribution of the subpopulations (<10 µm^2^ (**B**); 10–100 µm^2^ (**C**); >100 µm^2^ (**D**)) was independent of the underlying material surface. Data are presented as median (IQR). Statistics: two-way ANOVA (considering material effects of each volunteer) within ECMO-GFs and ECMO-HEs (independent analysis). *p*-values indicate significant differences among the materials. (**A**) ***; *p* < 0.001 compared to GF-PMP; §§§, *p* < 0.001 compared to GF-Hilite. Pairwise comparisons of HE materials are indicated. a, *p* < 0.001: comparison between GF and HE from Hilite-MLs. (**B**,**D**) Pairwise comparisons between GF and HE materials are indicated. a, *p* = 0.005: comparison between GFs and HEs from Nautilus-MLs (**C**,**D**).

Cytosceletal staining of platelets with rhodamine-phalloidin allowed the visualization of platelet adhesion on different ECMO material surfaces (Figure 2). Since GFs from different MLs are made of the same base material (PMP), changes in platelet adhesion (Figure 2A, left column) demonstrated coating effects. As shown in Figure 2A, surface coverage was highest on uncoated GFs [GF-PMP, 12 (7–19)%] as well as on X.ELLENCE-coated GFs [GF-Hilite, 13 (8–19)%, *p* = 0.330]. Platelet coverage on GF-PMP (or GF-Hilite) was significantly higher compared to all other GF coatings (*p* < 0.001). There was no difference regarding platelet coverage comparing the GFs that were coated with Bioline (GF-PLS), Balance (GF-Hilite) and PHISIO (GF-EOS). An isolated comparison of different HE materials/coatings (Figure 2A, right column) showed significantly higher platelet coverage on HE-Hilite compared to HE-Nautilus (*p* = 0.029). This was a coating effect, since both HEs were made of polyethyleneterephthalate (Table S1). Furthermore, X.ELLENCE-coated GFs (GF-Hilite, made of PMP) were covered with significantly more platelets compared to X.ELLENCE-coated HEs (HE-Hilite, made of polyethyleneterephthalate) (*p* < 0.001). This was an effect of the base material.

The particle size distribution of adherent platelets is shown in Figure 2B–D. Small aggregates (10–100 µm^2^) were predominant (independent of the test material) (50–60% of total particle numbers), while the smallest particles (<10 µm^2^) accounted for only 10–20%. The size distribution of particles on ECMO-GFs was not different for small and macro aggregates (Figure 2C,D), while a statistical difference was shown for the smallest particles (highest values for GF-PMP compared to GF-Nautilus, *p* = 0.004). However, due to the low proportion of the smallest particle sizes (<20%) and high variance of the data, the significance level is questionable. The size distribution on the HEs was different, with significantly more large particles on the X. ELLENCE-coated HEs (HE-Hilite) compared to the Balance-coated HEs (HE-Nautilus) and vice versa for the smallest particles. HE-PLS and HE-Hilite were comparable. Furthermore, the size distribution on HEs and GFs within the Nautilus ML was significantly different (fewer large particles on the HEs) (coating effect).

## 4. Discussion

In the present study, static hemocompatibility assays were used to test material-induced effects of naïve material surfaces [gas-exchange fibers (GFs), heat-exchange fibers (HEs)] from different commercially available ECMO MLs (with different antithrombogenic surface coatings) on the response of red blood cells (RBCs) (hemolysis) and isolated platelets (adhesion, activation). While no significant hemolysis or activation of circulating platelets was observed, platelet adhesion depended on the type of surface coating. Uncoated GF-PMP and GF-Hilite (X.ELLENCE-coating) presented significantly higher platelet coverage compared to GF-PLS (Bioline-coating), GF-Nautilus (Balance-coating) or GF-EOS (PH.I.S.I.O-coating).

Blood-contacting medical devices were tested for hemolytic activity [19]. The static test is based on the detection of erythrocyte lysis that is induced by contact, leachables, toxins, metal ions, surface charge or other causes of erythrocyte lysis [6,7]. Respective data for individual ECMO materials are not currently available. In the present study, it was found that different types of ECMO materials (GFs made of PMP, different HE materials, different antithrombogenic coatings) showed a mean hemolysis rate below 5%, being classified as non-hemolytic, aligning with ISO 10993-4:2017 and ASTM F756-00(2000) [20,21]. Therefore, we concluded that the clinically observed hemolysis on ECMO (with an incidence of 0–41%) is not due to a material-induced effect, but to increased shear forces [23,24,25,26,27] or disease-related factors [28,29]. One impressive hemolytic episode on ECMO is the sudden release of free hemoglobin after occlusion of the running pump head (pump head thrombosis)—the RBCs within the clot are destroyed by the continuing pump and free hemoglobin is released into the blood [8,25,30]. High concentrations of free hemoglobin in the blood can induce toxicity, alter the kidney function and cause thrombotic complications within the device [8,24,30].

Platelet activation is another important hemocompatibility test [5,16,17]. Platelet activation through biomaterials occurs via adhesion to adsorbed proteins and indirectly through biomaterial-induced activation of coagulation and other systems [6,7]. Besides platelet adhesion onto the biomaterial surface (see below), circulating platelets also come into contact with the biomaterial and remain in the soluble phase. Under in vivo conditions, these cells circulate in the blood stream and can induce both inflammatory and coagulation responses throughout the body. In the present study, a simple and rapid static platelet incubation model was used [17,31]. ECMO materials were incubated with PRP, and the resulting platelet activation of the circulating cells was quantified using FACS analysis (upregulation of membrane-bond CD62P, GPIIb/IIIa and GPIIIa; increased fibrinogen binding) [32]. It was shown that contact between ECMO materials and platelets did not result in an upregulation of membrane receptors or an alteration in their fibrinogen-binding activity. Therefore, we assumed that neither the artificial base materials (PMP, polyurethane, polyethylene terephthalate) nor the type of antithrombotic surface coating induced platelet activation. The static culture condition was a major limitation of this platelet model. However, it was shown that non-physiological shear stress induced platelet activation in blood-contacting medical devices, with a significant increase in the proportion and the MFI of CD62P and PAC-1-positive cells [27,33]. Both receptors were found in thrombotic deposits on gas-exchange fibers from used MLs, and platelet–leukocyte aggregates (PLAs) with increased expression of CD62P were also detected on the surface of the GFs far away from thrombotic aggregates [34,35]. High expression of CD62P on the platelet surface and high levels of PLAs were also found in blood samples from ECMO patients [36]. The underlying mechanism remained unclear.

Platelet adhesion is an essential parameter for verifying the hemocompatibility of a biomaterial [6,7]. In the clinical setting, when blood comes into contact with foreign surfaces in the ECMO circuit, plasma protein adsorption rapidly occurs, accompanied by the activation of circulating platelets and cell adhesion on the ECMO surfaces [6,7]. The static adhesion assay included an essential protein adsorptions step, since platelets were diluted in PPP, which contains all plasma proteins, followed by the subsequent platelet adhesion step. This test is a simple and cost-effective method for demonstrating the isolated effect of a multitude of surface and coating characteristics on platelet adhesion, allowing for semi-automated quantification. After adherence and fixation, cells were only stained with the fluorescent dye only, without further processing procedures, in order to ensure realistic cell adhesion [37]. The sample preparation of other test methods such as scanning electron microscopy often results in the loss of adherent cells. Despite its simplicity, the adhesion test used in the present study showed significant differences in the coating strategy (of materials with the same base material), as well as in the base material used for materials with the same coating. Uncoated PMP GFs showed good gas exchange performance but limited hemocompatibility [38], which could be improved after surface modification, such as the polymerization of phosphorylcholine [10,38,39]. Analogous to these data, in the present study, platelet adhesion was highest for uncoated PMP-GFs compared to the same GFs with bioactive (Bioline) as well as biopassive (Balance, Physio) coatings. One surprising finding was the high level of platelet binding on X.ELLENCE-coated Hilite-GFs, which was comparable to that observed on uncoated GFs. Both the Bioline and the X.ELLENCE coatings are bioactive coatings based on the multifaceted coating of albumin and heparin. They only differed in the bonding technique. Obviously, the binding of heparin to ATIII to catalyze AT-mediated inhibition of clotting factors such as thrombin and FXa was disturbed when using X.ELLENCE coating. Both GF-Hilite and GF-PLS originated from native and unexpired oxygenators and were prepared at the same time. However, it cannot be ruled out that the activity of the X.ELLENCE coating was impaired during storage. The increased platelet adhesion on the HE-Hilite compared to the HE-Nautilus (with the same base material—polyethyleneterephthalate) is a further indication for the reduced functionality of the X.ELLENCE coating. The improved hemocompatibility of bioactive surface coatings (such as Bioline) was in agreement with other studies [12,13]. Bioline-coated oxygenator surfaces reduced fibrinogen adsorption and GPIIb-IIIa binding, resulting in reduced platelet adhesion and activation [13]. Furthermore, PMP-surface coatings with phosphorylcholine also reduced platelet adherence and lowered the risk of blood clotting [38]. The authors speculated that surface coatings reduced the hydrophobic properties of uncoated PMP-GFs, reducing protein adsorption and platelet adhesion. Biopassive coatings such as Balance Biosurface, which contains negatively charged sulphonated polymers such as polyethylene oxide, increased the hydrophilicity of PMP, resulting in decreased protein adsorption and platelet adhesion [10,40].

Translating our in vitro results to the clinical setting is critical. In 2014, our research group demonstrated, in a large patient cohort, that the frequency of a system exchange (about 30%) was independent of the ECMO system [8,9]. However, the Hilite-MLs were mainly exchanged electively due to device-related coagulation disorder (57%), compared to the PLS-MLs (36%). To date, no data are available on coagulation disorders or the underlying exchange reasons when using Nautilus-MLs. A prognosis for future clinical use cannot be made based on the available in vitro data or the retrospective study by Lubnow et al. [8]. None of the commercially available ECMO systems are suitable for long-term use or as a fully implantable artificial lung. New technologies in surface coatings, such as endothelial membrane mimetic anticoagulation strategies [17] and novel device geometries with improved flow design, are essential to allow long-term use with a reduced thrombotic response [16].

Platelets adhered onto the GFs and HEs in form of microaggregates (10–100 µm^2^), while single cells and larger aggregates were less prominent. Statistical differences, indicated in Figure 2B–D, were negligible. Differences were mainly shown for small particles. However, there was a high standard deviation, although the mean values were below 20%. Differences in particle size do not correspond to changes in the shape of the adherent platelets, which is a recognized marker for platelet activation. Alternative methods are scanning electron microscopy (SEM) or immunofluorescence techniques that allow for the characterization of adhered platelet morphology [41,42]. However, these methods are time-consuming and expensive and require special equipment. The respective analysis was not performed in the present study. Platelet surface coverage was less than 10%. Nevertheless, this represents material-induced cell adhesion and indicates a risk of reduced hemocompatibility. In the clinical situation, platelet count decreased after ECMO implantation, and it was speculated that the majority of the cells adhered onto the large PMP surfaces of the MLs [10,25]. The results of the present study support this hypothesis. High shear forces during ECMO therapy, due to the blood pump, could further enhance this effect. As a result, increased platelet density and high CD62P expression were observed in thrombotic deposits on the surface of GF from used MLs, as well as in the clot-free areas in the form of PLAs [34,35].

Limitations: This is an in vitro study designed to assess the hemocompatibility of clinically established PMP gas-exchange fibers within newly developed, commercially available MLs. The static adhesion model used evaluated the interaction of platelets with the surface properties; however, visualization of cell shape as an indicator for platelet activation was not possible. In addition, this test did not analyze the effect of shear forces, which are essential contributors to ECMO-induced thrombosis. However, the significant differences in platelet adhesion were observed based on independent experiments in which all ECMO materials were incubated with platelets from seven different donors. Additional research using dynamic flow studies and extended duration tests is necessary to obtain more information about the essential effects of shear stress and prolonged blood contact with artificial surfaces on thrombus formation during ECMO therapy [8].

## 5. Conclusions

In vitro hemocompatibility testing with simple static culture models resulted in meaningful results. Circulating platelets remained non-activated after contact with any uncoated or coated ECMO material. Instead, commercially available bioactive and biopassive coatings reduced platelet adhesion by a factor of four compared to uncoated PMP GFs. One exception was the X.ELLENCE coating from GF-Hilite, which showed unexpectedly high platelet adhesion—potentially due to different bonding techniques. Furthermore, within the Hilte-ML, material effect was also observed—GFs exhibited higher platelet adhesion compared to the HEs made of polyethyleneterephthalate. In summary, this study confirms the good hemocompatibility of GFs and HEs under static conditions. However, X.ELLENCE coating was an exception, demonstrating higher platelet adhesion. The latter is not clinically representative. Additional hemocompatibility tests are necessary to assess the increased platelet adhesion observed on the materials from the Hilite-ML.

## Data Availability

The corresponding data are included in the article/Supplementary Materials, and further inquiries about this study can be directed to the corresponding author.

## Supplementary Materials

The following supporting information can be downloaded at https://www.mdpi.com/article/10.3390/biomedicines13102323/s1. Figure S1: Preparation process of test samples from a commercially available membrane lung (representative example: EOS membrane lung). Figure S2: Quantification of platelet density on the surface of gas fibers using ImageJ software. Figure S3: Representative flow cytometric analysis of material-induced expression of platelet activation markers. Figure S4: Representative FACS analysis of material-induced fibrinogen binding activity of platelets. Table S1: Coatings and material of commercially available membrane lungs (MLs). References [43,44,45,46,47] are cited in the Supplementary Materials.
