# Supplementary material for: Material-Induced Platelet Adhesion/Activation and Hemolysis of Membrane Lung Components from Extracorporeal Membrane Oxygenation
Source: Biomedicines. 2025 Sep 23;13(10):2323. doi: 10.3390/biomedicines13102323 (PMC12561393; doi:10.3390/biomedicines13102323)
Supplement: Supplementary file 1 [file biomedicines-13-02323-s001.zip › biomedicines-3712055-supplementary.pdf]

biomedicines

MDPI

Material-induced platelet adhesion/activation and hemolysis of Membrane Lung Components from Extracorporeal Membrane Oxygenation

Supplementary material

Antithrombogenic surface coatings – Mechanism of action of coated proteins and water-soluble polymers

Heparin binds to and accelerates the activity of antithrombin III (ATIII). The binding changes the structure of AT and accelerates the AT-mediated inhibition of clotting factors, including thrombin (FIIa) and Factor Xa. However, the anticoagulant performance is diminished and even lost due to a reduction in reactive sites, competitive protein adsorption, and chemical degradation or leaching off of the immobilized heparin [1].

Albumin is a passivating agent. It is the main protein of blood plasma and, due to its lack of peptide sequences for interaction with coagulation and complement system and cells, is not involved in blood coagulation and immune responses. Furthermore, steric repulsion by bound albumin is suggested for the prevention of protein adsorption and platelet adhesion [2].

Phosphorylcholine (PC) is a neutral, zwitterionic phospholipid that composes the surface of the lipid bilayer of cell membranes. PC coatings have been developed to modify the surface charge and hydrophilicity of foreign polymers to resemble that of endothelial cells [3]. In addition to serving as a physical barrier, phospholipids participate in cell-to-cell communication through alteration in charge and composition [4]. The hydrophilic characteristics form a surface hydration buffer to limit protein adsorption.

Polyethylene oxide (PEO) is a synthetic polymer surface that forms a hydration layer, creating a hydration barrier between the surface and the blood, which resists adsorption of plasma proteins to some extent and inhibits platelet interactions [5].

References

1. Biran, R.; Pond, D. Heparin coatings for improving blood compatibility of medicl devices. *Adv Drug Deliv Rev.* **2017**, 112, 12-23. doi: 10.1016/j.addr.2016.12.002.
2. Amiji, M.; Park, K. Surface modification of polymeric biomaterials with poly(ethylene oxide), albumin, and heparin for reduced thrombogenicity. *Biomater. Sci. Polym. Ed.* **1993**, 4, 217-234. doi: 10.1163/156856293x00537.
3. Maul, T.M.; Massicotte, M.P.; Wearden, P.D. ECMO biocompatibility: surface coatings, anticoagulation, and coagulation monitoring. Extracorporeal Membrane Oxygenation-Advances in Therapy: InTechOpen; **2016**.
4. Koseoglu, S.; Meyer, A.F.; Kim, D.; Meyer, B.M.; Wang, Y.; Dalluge, J.J.; Haynes C.L. Analytical characterization of the role of phospholipids in platelet adhesion and secretion. *Anal Chem.* **2015,** 87, 413-421. doi: 10.1021/ac502293p.
5. Cao, L.; Chang, M.; Lee, C.Y.; Castner, D.G.; Sukavaneshvar, S.; Ratner, BD.; Horbett, T.A. Plasma-deposited tetraglyme surfaces greatly reduce total blood protein adsorption, contact activation, platelet adhesion, platelet procoagulant activity, and in vitro thrombus deposition. *J. Biomed. Mater. Res. A.* **2007**, 81, 827-837. doi: 10.1002/jbm.a.31091.

Supplementary Figures

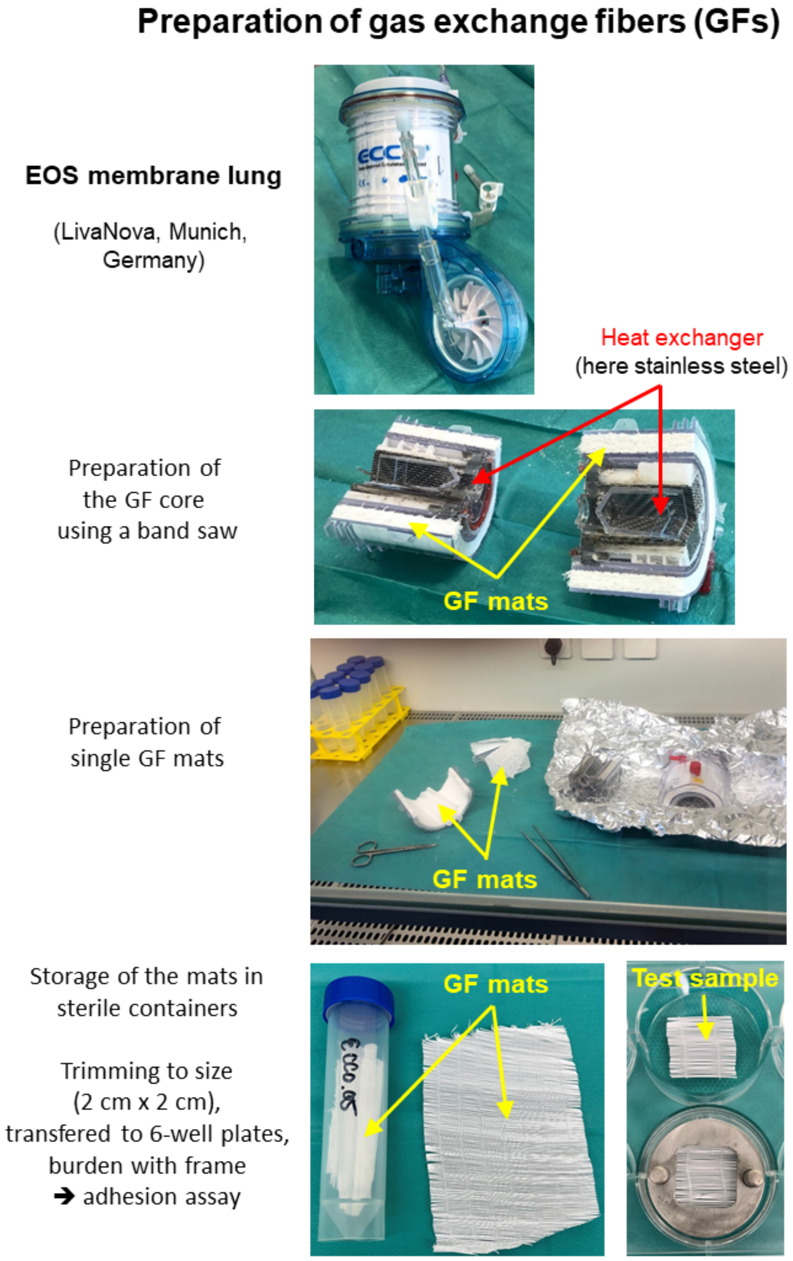

Figure S1. Preparation process of test samples from a commercially available membrane lung (representative example: EOS membrane lung).

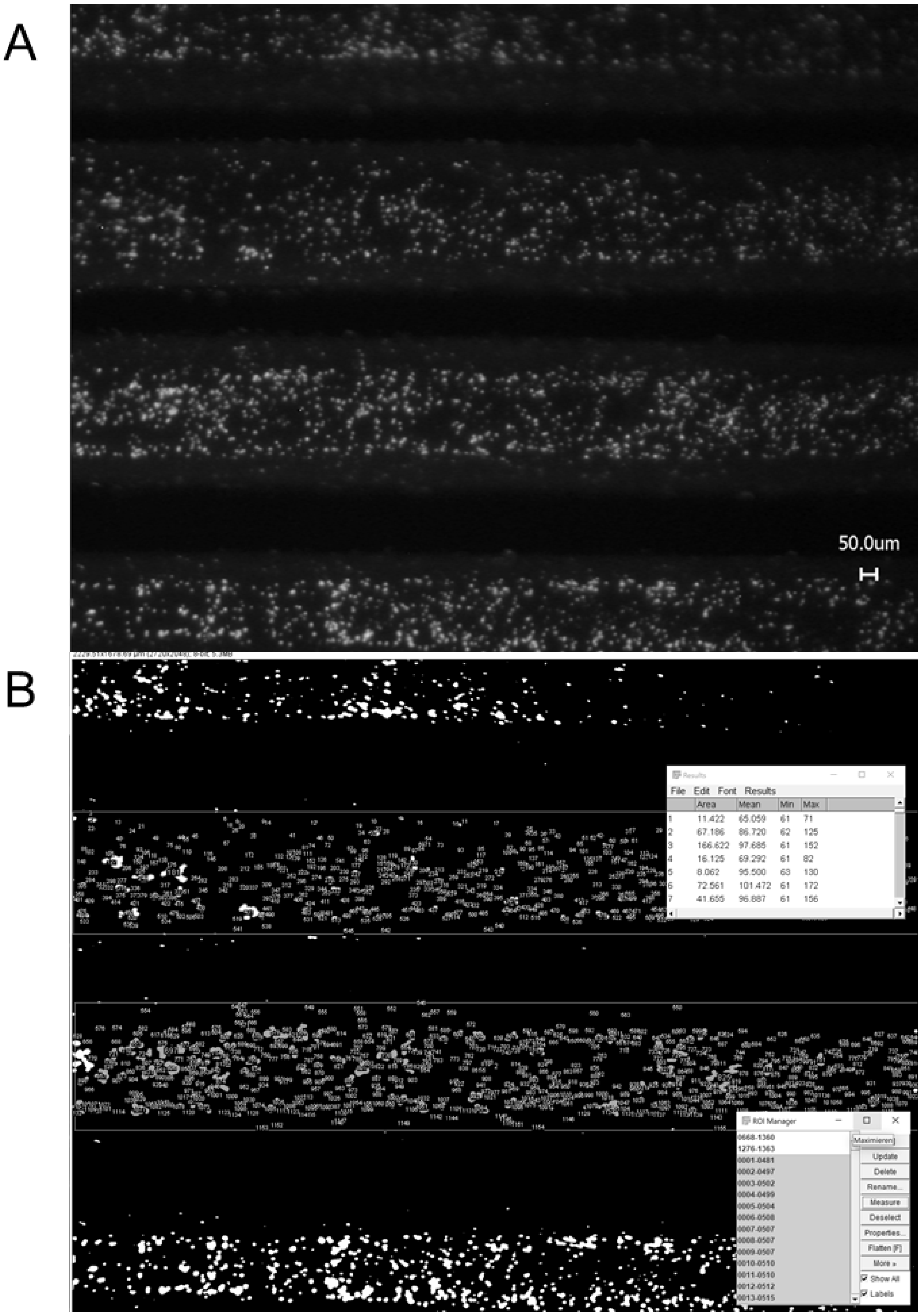

Figure S2. Quantification of platelet density on the surface of gas fibers using ImageJ software. Images from rhodamine-phalloidin stained adherent platelets on the surface of test samples were imported into ImageJ program and converted to grayscale (A). Individual region of interests (ROI) were selected along two GF (total area of ROI, 1.53 mm^2) and one HE fibers (total area of ROI, 0.77 mm^2) (B). The area in mm^2 was calculated using the scale bar (50 μm). The single particle areas, the sum of the area of all particles within the ROIs, the total cell density as well as the proportion of different particle sizes relative to the total particle count was calculated.

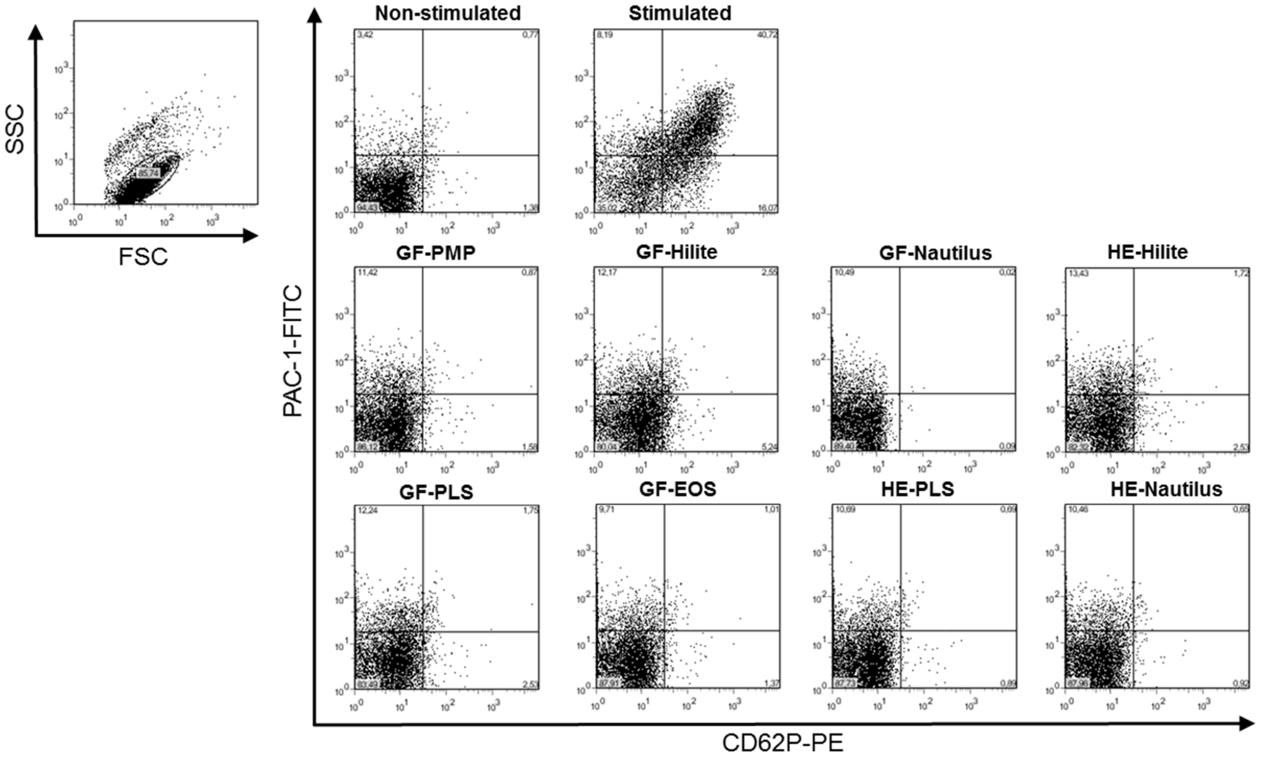

Figure S3. Representative flow cytometric analysis of material-induced expression of platelet activation markers. Platelets in the supernatants of different ECMO-materials were stained with PAC-1-FITC and CD62-PE antibodies and measured by FACS. FSC vs SSC localized platelets (left column). Gated platelets were visualized in a dotplot of CD62P-PE) vs PAC-1-FITC. The signal was subdivided into 4 quadrants to identify different subpopulations as shown for non-stimulated cells that were CD62P-/PAC-1-. In contrast, stimulation with ADP resulted in an increase in the frequency with higher median fluorescence intensity of double stained cells (CD62P+/PAC-1+). Stimulated (non-stimulated) cells were treated with ADP (buffer) without material contact. ECMO materials were gas fibers (GF) and heat exchange fibers (HE) from four different membrane lungs (PLS, Hilite, EOS, Nautilus) that represent different antithrombogenic surface coatings (Table S1). GF-PMP was the uncoated reference material.

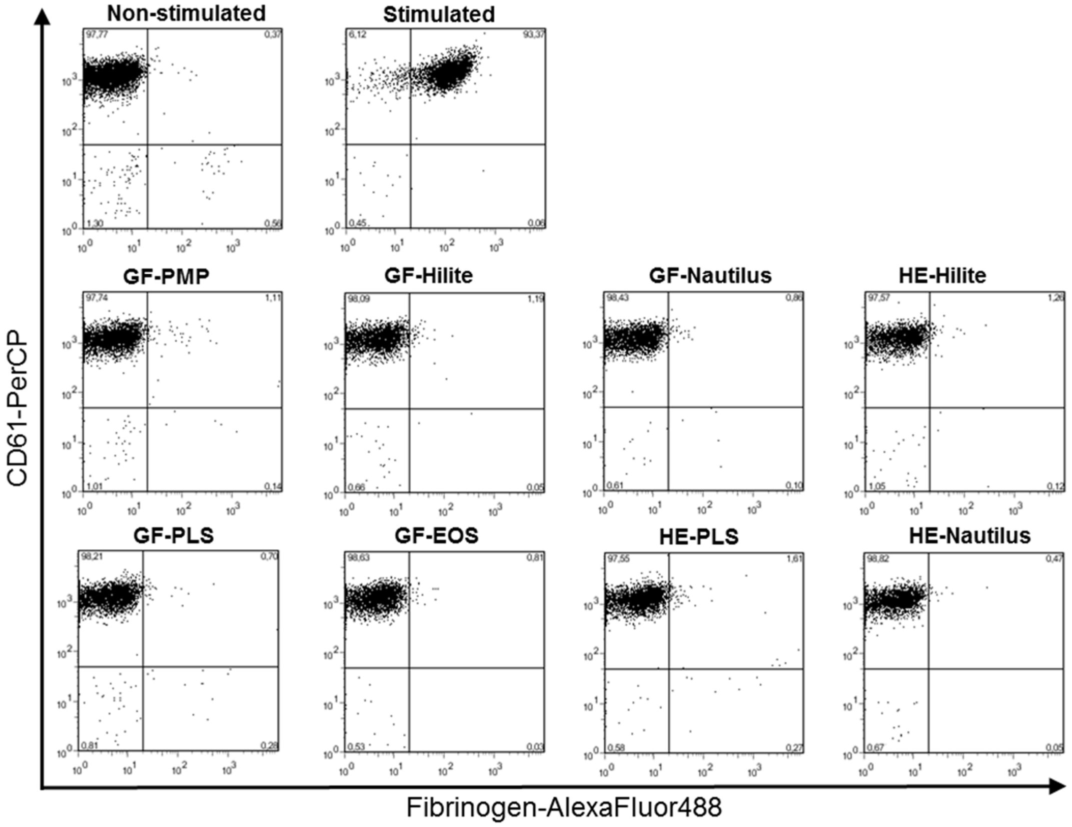

Figure S4. Representative FACS analysis of material-induced fibrinogen binding activity of platelets. Platelets in the supernatants of different ECMO materials were stained with fibrinogen-AlexaFluor488 and CD61-PerCP antibody and measured by FACS. FSC vs SSC localized platelets. Gated platelets were visualized in a dotplot of fibrinogen-AlexaFluor488 vs CD61-PerCP. The dotplot was subdivided into 4 quadrants to identify different subpopulations as shown for non-stimulated cells that were CD61+/fibrinogen-, while stimulation with ADP resulted in an increase in the frequency with higher median fluorescence intensity of double stained cells (CD61+/fibrinogen+). None of the ECMO materials induce an increase in the fibrinogen-binding activity. Stimulated (non-stimulated) cells were treated with ADP (buffer) without material contact. ECMO materials were gas fibers (GF) and heat exchange fibers (HE) from four different membrane lungs (PLS, Hilite, EOS, Nautilus) that represent different antithrombogenic surface coatings (Table S1). GF-PMP was the uncoated reference material.

Supplementary Tables

Table S1. Coatings and material of commercially available membrane lungs (MLs).

ML, type	GF, material	GF, surface area	HE, material	HE, surface area	Coating	Type of coating	Coating, mode of operation
PMP[a]	PMP	--	--	--	Uncoated	--	--
PLS[a]	PMP	1.8 m^2	Polyurethane	0.4 m^2	Bioline®	bioactive	Covalent bonds between heparin molecules and an albumin layer
Hilite 7000LT[b]	PMP	1.9 m^2	Polyethylene-terephthalate	0.45 m^2	X.EL-LENCE®	bioactive	Heparin covalently and ionically bound to immobilised albumin in several layers
Nautilus[c]	PMP	1.8 m^2	Polyethylene-terephthalate	0.3 m^2	Balance®	biopassive	Polyethylene oxide (PEO), integration of sulphate and sulphonate groups
EOS[d]	PMP	1.2 m^2	Stainless steel[e]	0.14 m^2	PH.I.S.I.O®	biopassive	Phosphorylcholin (PC)

GF, gas fibers; HE, heat exchange fibers. Manufacturer of MLs: [a] Getinge, Rastatt, Germany; [b] Fresenius Medical Care, Bad Homburg, Germany; [c] Medtronic, Meerbusch, Germany; [d] L,ivaNova, Munich; Germany. All GFs were of polymethylpentene (PMP). [e] HE from the EOS-ML was not tested. Details on mechanism of function of coating material see supplementary material.
